# Local inconsistency detection using the Kullback–Leibler divergence measure

**DOI:** 10.1186/s13643-024-02680-4

**Published:** 2024-10-17

**Authors:** Loukia M. Spineli

**Affiliations:** Midwifery Research and Education Unit, Hannover Medical School, Hannover, 30625 Germany

**Keywords:** Network meta-analysis, Consistency, Kullback–Leibler divergence, Information loss

## Abstract

**Background:**

The standard approach to local inconsistency assessment typically relies on testing the conflict between the direct and indirect evidence in selected treatment comparisons. However, statistical tests for inconsistency have low power and are subject to misinterpreting a *p*-value above the significance threshold as evidence of consistency.

**Methods:**

We propose a simple framework to interpret local inconsistency based on the average Kullback–Leibler divergence (KLD) from approximating the direct with the corresponding indirect estimate and vice versa. Our framework uses directly the mean and standard error (or posterior mean and standard deviation) of the direct and indirect estimates obtained from a local inconsistency method to calculate the average KLD measure for selected comparisons. The average KLD values are compared with a semi-objective threshold to judge the inconsistency as acceptably low or material. We exemplify our novel interpretation approach using three networks with multiple treatments and multi-arm studies.

**Results:**

Almost all selected comparisons in the networks were not associated with statistically significant inconsistency at a significance level of 5%. The proposed interpretation framework indicated 14%, 66%, and 75% of the selected comparisons with an acceptably low inconsistency in the corresponding networks. Overall, information loss was more notable when approximating the posterior density of the indirect estimates with that of the direct estimates, attributed to indirect estimates being more imprecise.

**Conclusions:**

Using the concept of information loss between two distributions alongside a semi-objectively defined threshold helped distinguish target comparisons with acceptably low inconsistency from those with material inconsistency when statistical tests for inconsistency were inconclusive.

**Supplementary Information:**

The online version contains supplementary material available at 10.1186/s13643-024-02680-4.

## Background

The medical research landscape has undergone unprecedented growth, characterised by a surge of primary and secondary research of various scientific quality and novelty investigating several healthcare treatments of different complexity [1]. The evergrowing scientific evidence in quantity and complexity has led to a paradigm shift in the evidence synthesis methods, establishing network meta-analysis (NMA), an extension of pairwise meta-analysis, as the statistical tool to address research questions on multiple treatments for health technology assessments, guideline development, and clinical research [2, 3]. Fast-paced advances in the methodology of NMA and software availability over the past decades have driven the rapid increase in publications of systematic reviews with NMA [2–4].

Indirect evidence comprises the central component of the NMA methodology [5, 6]. It refers to evidence for a pairwise comparison (e.g. C versus A) informed by different sets of studies sharing one or more common comparators (e.g. B versus A and C versus B) through the consistency equation (the effect of C versus A equals the effect of B versus A and the effect of C versus B) [7]. The indirect estimate can yield reliable information about the compared treatments, provided that the evidence contributing to the consistency equation is similar concerning important effect modifiers [8]. Access to direct evidence for that comparison allows for assessing whether the indirect evidence represents the direct evidence. Lack of agreement between different sources of evidence has been termed inconsistency and can compromise the quality of conclusions [7].

Inconsistency assessment has received great methodological attention for pinpointing locations in the evidence network where the different sources mismatch, requiring immediate attention [2, 9–11]. Different methods have been developed to evaluate inconsistency, widely distinguished into local and global methods [12]. Methods for local inconsistency evaluation have received relatively more attention from systematic review authors and methodologists, probably for being intuitively more appealing and long-established since the introduction of NMA [2, 12, 13]. Local inconsistency evaluation aims at closed loops of evidence in the network (there is direct and indirect evidence for the involved treatment comparisons), where specific treatment comparisons are targeted to disentangle the direct from the indirect effect and calculate their difference, known as inconsistency [12]. Typically, the selected comparisons are inspected for statistically significant inconsistency manifested as a two-sided *p*-value of the *Z*-test that does not exceed a significance level (usually at 5% or 10%) or 95% confidence (credible) intervals that exclude a zero inconsistency [12, 14]. We call this framework ‘standard decision-making’.

Nevertheless, undue reliance on these measures may mask a material inconsistency when the comparisons in the loops are insufficiently informed, and between-study variance is substantial, as there is likely low power to detect a statistically significant inconsistency [15]. On the other hand, researchers often misinterpret a statistically non-significant inconsistency as proof of consistency. Clearly, a different route is needed when *interpreting* results from local inconsistency evaluation to protect against (1) missing material inconsistency due to low-power issues and (2) misinterpreting statistically nonsignificant inconsistencies as evidence of consistency, a necessity also echoed by other authors and pertains to global inconsistency assessment, as well [16].

We draw inspiration from the Kullback–Leibler divergence (KLD) measure to set up a novel and straightforward *interpretation* framework for local inconsistency evaluation that (1) shifts from *p*-values and confidence (credible) intervals to the whole distribution of the estimated direct and indirect effects and (2) semi-objectifies the thresholds selected to aid interpretation. The KLD is a well-established measure of entropy that quantifies information loss between two distributions, D for the observed data and A as an approximation of D, by using distribution A rather than D [17]. A similar analogy can be transferred to the NMA framework when assessing inconsistency locally: how much information is lost when the indirect effect replaces the direct effect, and vice versa, for a selected comparison. Minimum information loss would imply low inconsistency that may not threaten the validity of NMA results. A carefully selected threshold is required to define minimum information loss, and access to relevant empirical evidence may play a pivotal role in developing an intuitive decision threshold.

The rest of the article is structured as follows. We first present three motivating examples from methodological articles on local inconsistency evaluation using the node-splitting and back-calculation approaches [14, 18]. Then, we introduce our proposed interpretation framework for local inconsistency evaluation based on the KLD measure. We demonstrate our framework using the motivating examples. Finally, we discuss our framework, juxtaposing the evidence from the relevant published literature, and conclude with the usefulness of the proposed framework when interpreting the local inconsistency evaluation results.

### Motivating examples

**Fig. 1:**
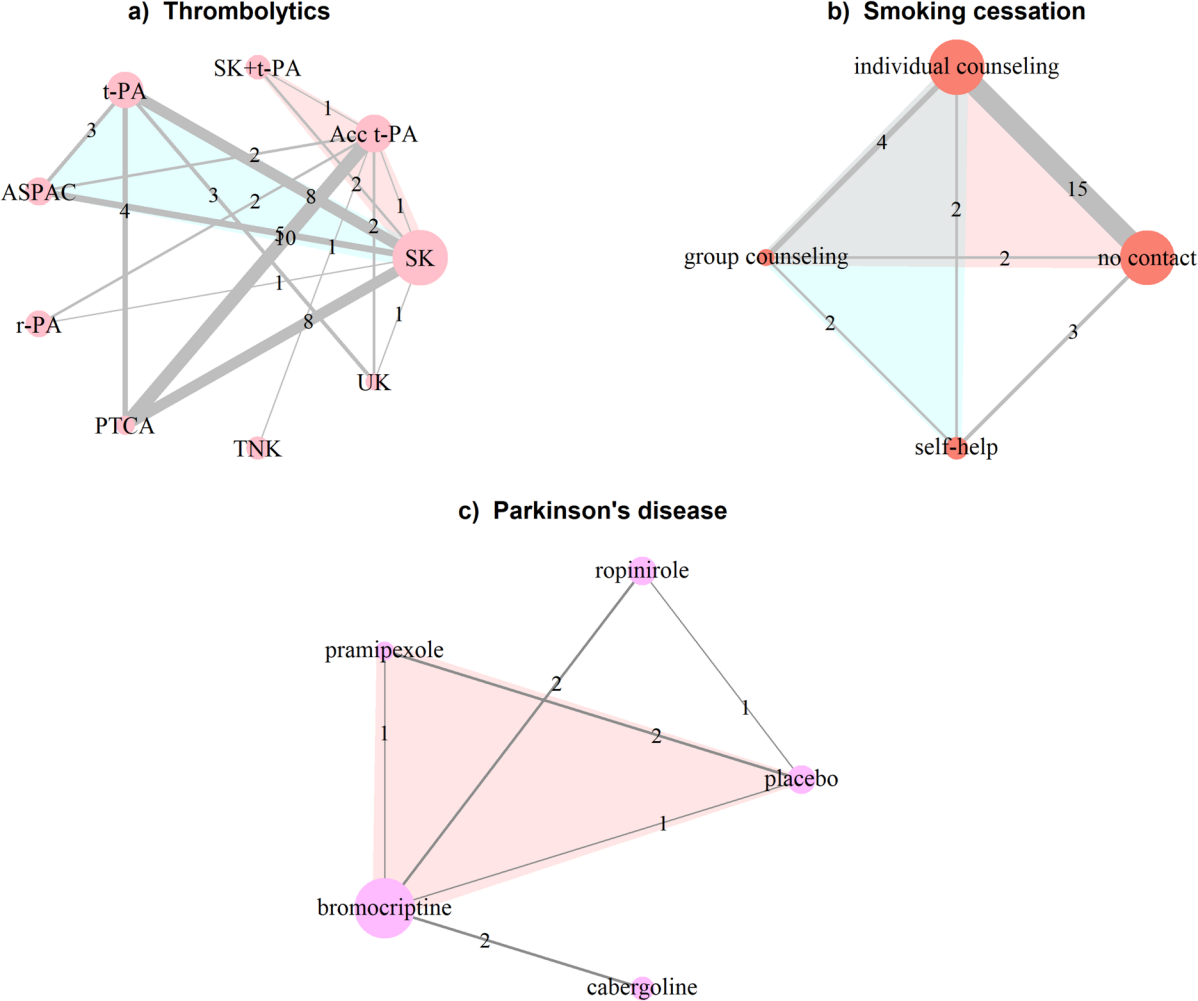
Network plots on **a** thrombolytics (first example) [19, 20], **b** smoking cessation (second example) [21], and **c** Parkinson’s disease (third example) [22]. Each node refers to a treatment and each edge to an observed (direct) comparison. The nodes’ size and the edges’ thickness are proportional to the number of randomised participants in the respective treatments and the number of studies investigating the respective comparisons. Numbers on the edges refer to the number of studies. Coloured loops indicate multi-arm studies. Acc t-PA, accelerated alteplase; t-PA, alteplase; ASPAC, anistreplase; PTCA, percutaneous transluminal coronary angioplasty; r-PA, reteplase; SK, streptokinase; SK + t-PA, streptokinase plus alteplase; TNK, tenecteplase; UK, urokinase

The first example is the well-known network of 50 studies (48 two-arm and 2 three-arm) comparing 8 thrombolytic treatments and angioplasty administered after acute myocardial infarction (thrombolytic network): streptokinase (SK), alteplase (t-PA), accelerated alteplase (Acc t-PA), streptokinase plus alteplase (SK + t-PA), reteplase (r-PA), tenecteplase (TNK), percutaneous transluminal coronary angioplasty (PTCA), urokinase (UK), and anistreplase (ASPAC) [19, 20] (Fig. 1a). The outcome is binary and refers to death in 30 or 35 days. Dias et al. [14] used a fixed-effect model to apply two local inconsistency methods, the back-calculation, and node-splitting approaches. The authors reported the posterior mean and standard deviation of NMA, direct and indirect log-odds ratios (OR), the inconsistency estimate, and the two-sided Bayesian *p*-values for each selected comparison (split node) (Table 2 in [14]).

Another well-known dataset is the network of smoking cessation treatments, which comprised the second example [21]. The network includes 24 studies (22 two-arm and 2 three-arm) investigating different sets of 4 smoking cessation counselling programmes, including no intervention, self-help, individual, and group counselling (Fig. 1b)). The outcome is binary and refers to smoking cessation (yes or no) at 6 to 12 months. Dias et al. [14] applied the node-splitting approach using a random-effects model. The authors reported the results in line with the first motivating example (Table 3 in [14]).

The third example is a network of seven studies (six two-arm and one three-arm) investigating four dopamine agonists and a placebo for Parkinson’s disease [22]: pramipexole, ropinirole, bromocriptine and cabergoline (Fig. 1c). The outcome is the average off-time period where Parkinson’s symptoms are out of control. The dataset was used by van Valkenhoef et al. [18] to illustrate their approach to automatic node splitting. They reported a forest plot with the posterior NMA, direct and indirect mean differences (MD), and the two-sided Bayesian *p*-value for inconsistency for the split comparisons (Fig. 6 in [18]).

## Methods

### Kullback–Leibler divergence measure for the inconsistency extent

In the Bayesian framework, a non-informative prior normal distribution is assigned to the treatment effects of comparisons with the reference treatment (called ‘basic parameters’ in the literature [7]). For the relative measures, this prior distribution applies to the logarithmic scale. Since these comparisons’ prior and posterior distributions are conjugate, their posterior distribution is also normal. Hence, the posterior distribution of indirect estimates is normal for being a linear combination of the basic parameters through the consistency equation [7]. At the same time, in the frequentist framework, the estimated direct and indirect effects are typically assumed to follow a normal distribution (again, for the relative measures, this assumption applies to the logarithmic scale). Therefore, we consider the KLD measure for two normal distributions defined using the following formula [17]:

1 $${D}_{D,I}^{j}=\frac{1}{2}\left[{\left(\frac{{\widehat{s}}_{D}}{{\widehat{s}}_{I}}\right)}^{2}+\frac{{\left({\widehat{\mu }}_{D}-{\widehat{\mu }}_{I}\right)}^{2}}{{\widehat{s}}_{I}^{2}}-1+ln\left(\frac{{\widehat{s}}_{I}^{2}}{{\widehat{s}}_{D}^{2}}\right)\right]$$

with ${D}_{D,I}^{j}$ implying the direct estimate (subscript D) is approximated by the indirect estimate (subscript I) for the *target comparison* $j$ (i.e. comparison with direct and indirect evidence selected to quantify inconsistency), ${\widehat{\mu }}_{D}$ and ${\widehat{\mu }}_{I}$ being the corresponding means in the frequentist framework or posterior means in the Bayesian framework, and ${\widehat{s}}_{D}$ and ${\widehat{s}}_{I}$ being the corresponding standard errors in the frequentist framework or posterior standard deviations in the Bayesian framework. We have dropped the comparison index $j$ in the abovementioned parameters for ease of presentation.

Then, the KLD measure of approximating the indirect estimate by the direct estimate is provided by the following:

2 $${D}_{I,D}^{j}=\frac{1}{2}\left[{\left(\frac{{\widehat{s}}_{I}}{{\widehat{s}}_{D}}\right)}^{2}+\frac{{\left({\widehat{\mu }}_{D}-{\widehat{\mu }}_{I}\right)}^{2}}{{\widehat{s}}_{D}^{2}}-1+ln\left(\frac{{\widehat{s}}_{D}^{2}}{{\widehat{s}}_{I}^{2}}\right)\right]$$

The average of ${D}_{D,I}^{j}$ and ${D}_{I,D}^{j}$ indicates the *average* information loss when approximating one estimate with the other for the target comparison $j$, denoted by ${D}^{j}$. We calculate as many ${D}^{j}$ as the number of target comparisons in a connected network with closed loops that are not informed exclusively from multi-arm studies. The ${D}^{j}$ should not be confused with a distance measure because it does not fulfil all the properties of a distance measure.

The probability densities of the distributions are the core element of the KLD measure, and the difference in their probability densities essentially determines the extent of information loss from approximating one distribution with the other. Intuitively, the more the distributions of the direct and indirect estimates overlap for a target comparison, the less information is lost (on average) when approximating one evidence source with the other. Hence, the smaller the ${D}^{j}$ value, the more likely to have low inconsistency. The KLD takes nonnegative values with ${D}^{j}=0$ indicating that both estimates’ distributions overlap perfectly; hence, the corresponding target comparison is not associated with inconsistency. This would be the ideal scenario; however, ${D}^{j}$ is more likely to be positive, raising the question of what constitutes acceptably low information loss and, thus, an acceptable inconsistency that does not threaten the conclusions.

### Setting the threshold of acceptably low inconsistency

Since the KLD measure for two normal distributions (and the byproduct ${D}^{j}$) is a function of the mean and variance of the estimated direct and indirect effects, it contains all information on their distributions and can be used to propose an intuitive decision rule to judge whether ${D}^{j}$ signals acceptably low or material inconsistency in the target comparison. We propose a reference threshold, adopting the *opinion elicitation* framework of Spiegelhalter et al. [23] regarding a plausible prior distribution for the between-study variance (${\tau }^{2}$) and translating it into the inconsistency framework.

Spiegelhalter et al. [23] described a case of two independent parameters, ${\theta }_{1}$ and ${\theta }_{2}$, following the same normal distribution with variance ${\tau }^{2}$, whose difference indicates the effect of a treatment relative to a control; hence, ${\theta }_{1}-{\theta }_{2}\sim N\left(0, 2{\tau }^{2}\right)$. Then, the absolute difference constrained to be above 0 would follow a half-normal distribution with scale parameter $\sqrt{2}\tau$: $\left|{\theta }_{1}-{\theta }_{2}\right|\sim HN\left(\sqrt{2}\tau \right)$ [23]. The median of that half-normal distribution is ${\Phi }^{-1}\left(0.75\right)\times \sqrt{2}\tau \cong 0.95\tau$ and represents the median difference between the maximum and minimum of a random pair $\left({\theta }_{1},{\theta }_{2}\right)$ on the absolute scale [24].

**Fig. 2:**
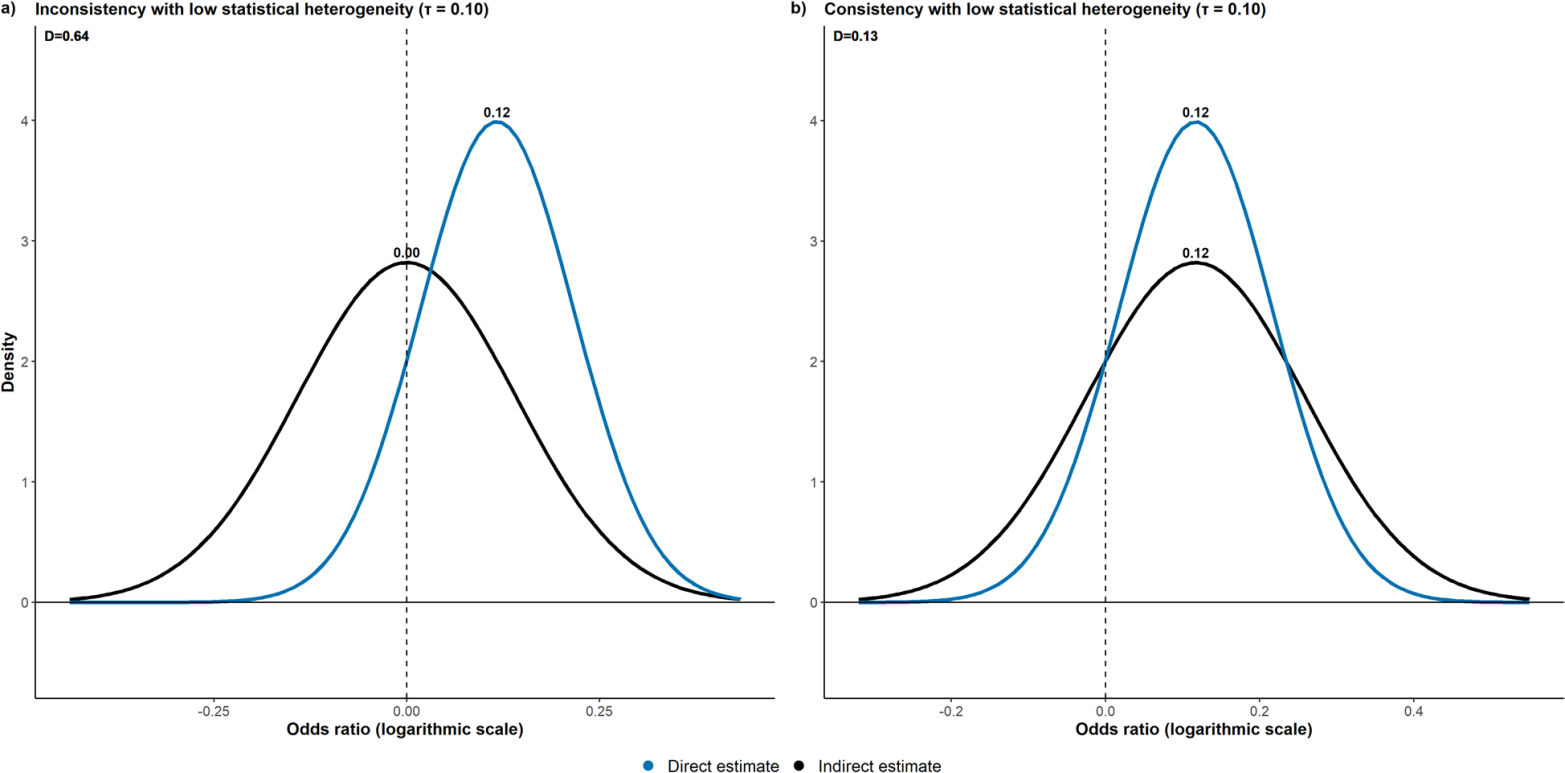
**a** Probability densities of the direct (blue line) and indirect (black line) log ORs for a fictional target comparison assuming ${\widehat{\mu }}_{D}-{\widehat{\mu }}_{I}=1.17\tau$ (inconsistency evidence) and $\tau =0.1$ (low statistical heterogeneity [23]), yielding an average information loss ($D$) of 0.64. **b** Probability densities of the direct (blue line) and indirect (black line) log ORs for a fictional target comparison assuming ${\widehat{\mu }}_{D}-{\widehat{\mu }}_{I}=0$ (consistency evidence) and $\tau =0.1$ (low statistical heterogeneity [23]), yielding an average information loss of 0.13 (stricter threshold)

Leveraging this framework for inconsistency would correspond to ${\theta }_{1}$ and ${\theta }_{2}$ be the direct and indirect effects that follow the same normal distribution but with variances ${\tau }^{2}$ and ${2\tau }^{2}$, respectively. Then, their difference would follow a normal distribution with zero mean and variance $3{\tau }^{2}$, describing the inconsistency between these two sources. Their absolute difference constrained to be above 0 would have a half-normal distribution with scale parameter $\sqrt{3}\tau$ and median equal to ${\Phi }^{-1}\left(0.75\right)\times \sqrt{3}\tau \cong 1.17\tau$ on an absolute scale, such as (standardised) mean difference and relative measures on the logarithmic scale. Replacing ${\widehat{\mu }}_{D}-{\widehat{\mu }}_{I}=1.17\tau$, ${\widehat{s}}_{D}=\tau$, and ${\widehat{s}}_{I}=\sqrt{2}\times \tau$ in Eqs. (1) and (2) and calculating their average, we obtain an average information loss of 0.64. Hence, ${D}^{j}<0.64$ implies acceptably low inconsistency for the target comparison $j$ on the absolute scale. Figure 2a illustrates the probability densities of the direct and indirect estimates that differ in their location by $1.17\tau$, assuming $\tau =0.10$, which implies low statistical heterogeneity [23]*.*

Note that we do not need to define $\tau$ as it is cancelled out in both equations. Regardless of $\tau$, the average information loss remains at 0.64 because the distributions overlap enough in the range of values despite their different locations (Fig. 2a). Hence, target comparisons with poorly overlapping ranges in the direct and indirect estimates will be penalised with an average information loss beyond the threshold. Considering a zero difference between the direct and indirect effects (i.e. ${\widehat{\mu }}_{D}-{\widehat{\mu }}_{I}=0$ in both equations) would yield a stricter threshold of 0.13, irrespective of $\tau$ value (Fig. 2b).

### Methods implementation and software

We used directly the results on the direct and indirect estimates for the target comparisons reported in the first two examples (thrombolytics and smoking cessation networks) and calculated the corresponding ${D}^{j}$ values. The third example (Parkinson ‘s disease) was analysed in the MD scale. We re-ran this example in the same scale using the default arguments of the gemtc R package [25] (as considered by the authors [18]) because the article did not provide the posterior standard deviation of the direct and indirect estimates.

The rnmamod R package [26] was used to calculate the ${D}^{j}$ for the target comparisons (kld_measure function), infer the magnitude of inconsistency as acceptably low or material for the threshold of 0.64 (kld_inconsistency or kld_inconsistency_user functions), and create all figures (via the former two functions). 

## Results

### Thrombolytics network (first example)

**Fig. 3:**
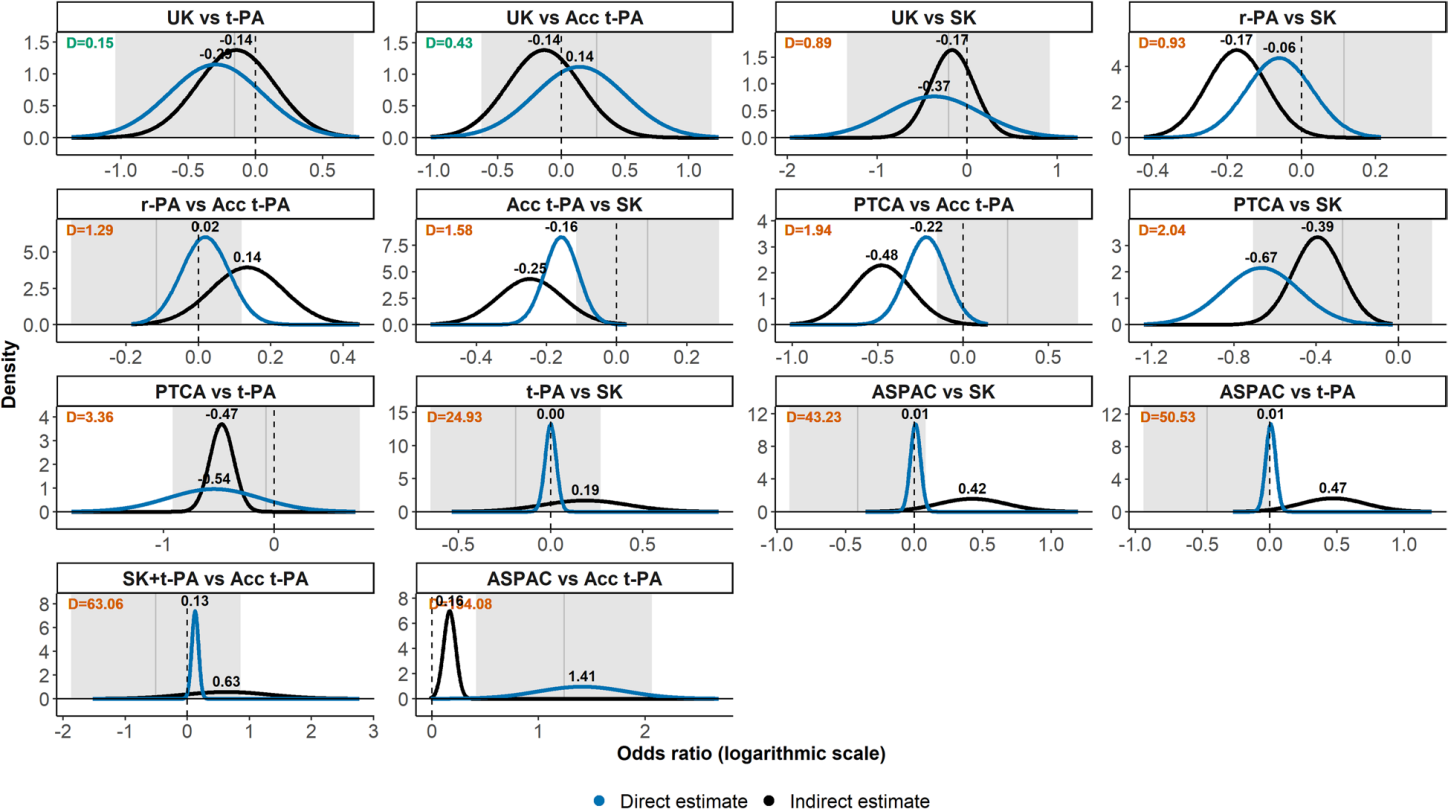
Posterior densities of the direct (blue line) and indirect (black line) log ORs for 14 target comparisons from the thrombolytics network (first example). The grey area and vertical line indicate the inconsistency’s 95% interval (approximated using the reported posterior mean and standard deviation) and posterior mean. The average information loss (${D}^{j}$) appear at the top left of each plot. The plots have been sorted in ascending order of the ${D}^{j}$ values. The *x*-axis and *y*-axis values vary across all plots. Green and orange ${D}^{j}$ values indicate acceptably low and material inconsistency. The threshold of 0.64 was employed

Figure 3 presents the posterior densities of the direct and indirect log ORs of 14 target comparisons obtained using the node-splitting approach (Table 2 in [14]). The grey area and vertical line refer to the inconsistency’s 95% interval and posterior mean in each target comparison. The plots have been sorted in ascending order of the ${D}^{j}$ values (the superscript has been dropped from the plots for simplicity). There was statistically significant inconsistency only for ASPAC versus Acc t-PA based on the 95% interval for inconsistency.

The target comparison UK versus t-PA had the lowest ${D}^{j}$ value at 0.15, below the threshold of 0.64, exhibiting acceptably low inconsistency (Fig. 3). The subsequent target comparison (UK versus Acc t-PA) had opposing conclusions at the posterior mean sign regarding the effectiveness of the compared treatments; however, the distributions had posterior means close to 0 and overlapped enough, yielding an average information loss close to but below the threshold.

For the remaining target comparisons, direct and indirect estimates yielded the same effectiveness conclusions (at the posterior mean sign); however, they differed substantially in the posterior mean and standard deviation in most target comparisons, exhibiting poor overlap in their posterior densities (especially the last six target comparisons) and, hence, a substantial average information loss (Fig. 3). ASPAC versus Acc t-PA had an enormous ${D}^{j}$ value at 134.08: the direct log OR was around nine times larger than the indirect log OR and seven times more imprecise, leading to an immense information loss due to no overlap of the posterior densities (Fig. 3).

**Fig. 4:**
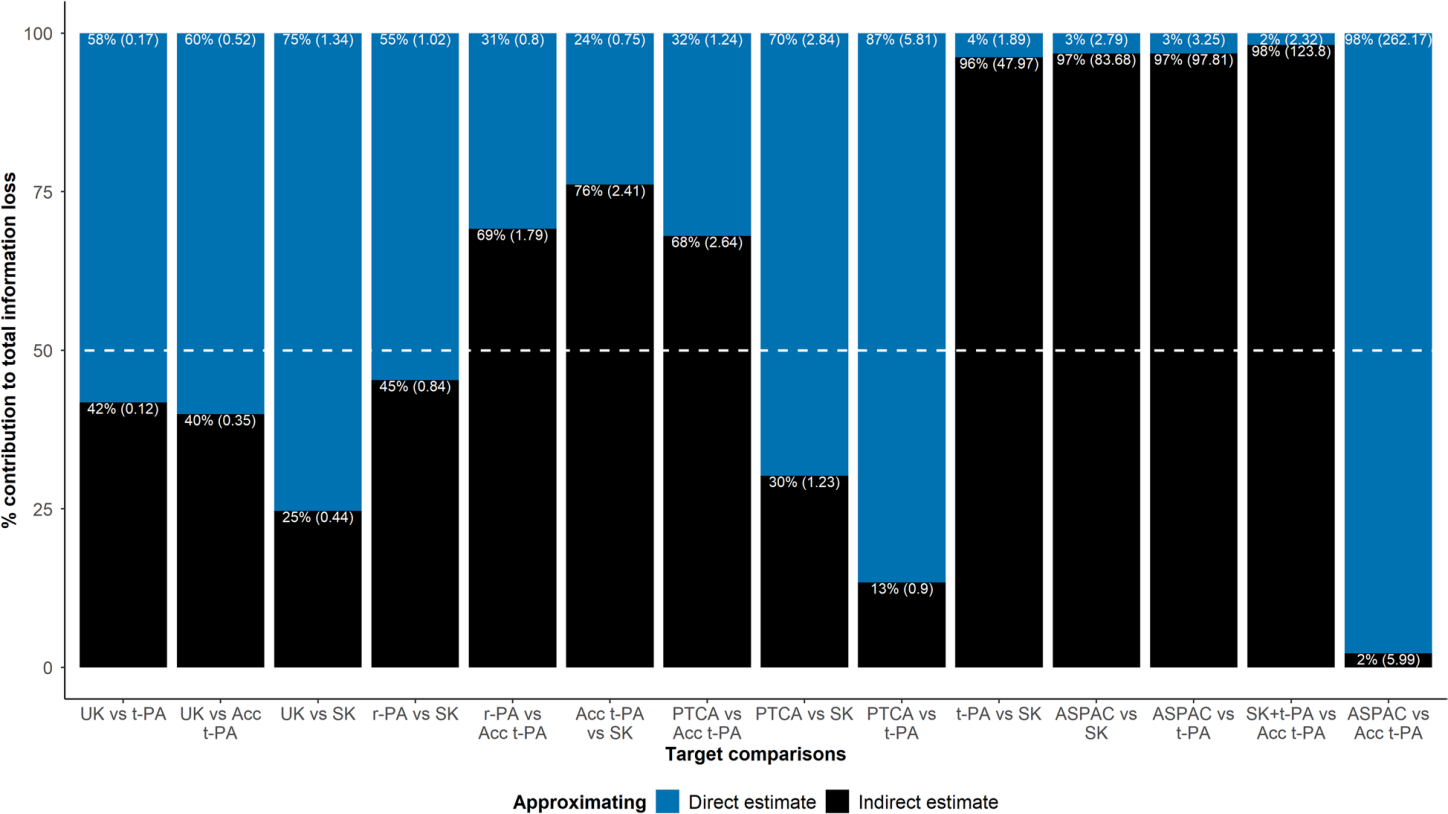
Bar plots with the percentage contribution of approximating direct posterior density (blue bars, ${D}_{D,I}^{j}$) and indirect posterior density (black bars, ${D}_{I,D}^{j}$) to their total information loss (${D}_{D,I}^{j}+{D}_{I,D}^{j}$) for each target comparison (*x*-axis) from the thrombolytics network (first example). Percentage contributions appear outside the parenthesis. The plots have been sorted in ascending order of the ${D}^{j}$ values. The ${D}_{D,I}^{j}$ and ${D}_{I,D}^{j}$ values appear in the parentheses

Figure 4 illustrates a bar plot with the percentage contribution of ${D}_{D,I}^{j}$ (blue bars) and ${D}_{I,D}^{j}$ (black bars) to the total information loss (${D}_{D,I}^{j}+{D}_{I,D}^{j}$) for each target comparison. The bars are sorted in ascending order of the target comparisons' ${D}^{j}$ values. The ${D}_{D,I}^{j}$ and ${D}_{I,D}^{j}$ values appear in the parentheses. In target comparisons with trivial overlapping of their direct and indirect densities (the last six target comparisons in Fig. 3), approximating an overly imprecise distribution contributed almost exclusively to the total information loss.

Additional file 1: Figure S1 presents the density plots for the results from the back-calculation (Table 2 in [14]), also pointing to material inconsistency in the network for the same target comparisons. Overall, the ${D}^{j}$ values were very similar to those from the node-splitting approach, except for ASPAC versus t-PA, SK + t-PA versus Acc t-PA, and ASPAC versus SK, where the ${D}^{j}$ values were notably smaller under the back-calculation approach for yielding indirect posterior densities much closer to the direct posterior densities, exhibiting a comparatively better overlapping.

### Smoking cessation network (second example)

**Fig. 5:**
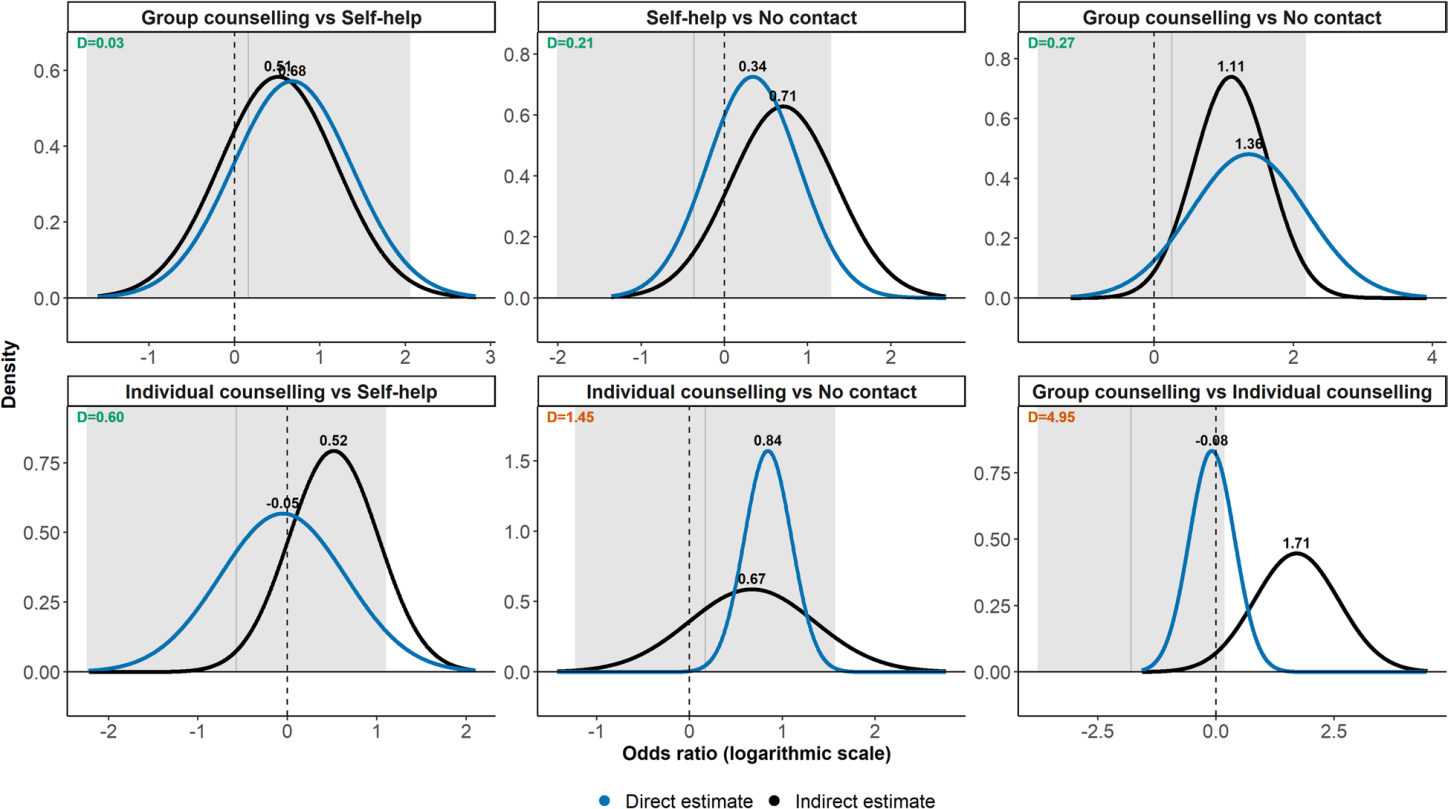
Posterior densities of the direct (blue line) and indirect (black line) log ORs for six target comparisons from the smoking cessation network (second example). The grey area and vertical line indicate the inconsistency’s 95% interval (approximated using the reported posterior mean and standard deviation) and posterior mean. The average information loss (${D}^{j}$) appear at the top left of each plot. The plots have been sorted in ascending order of the ${D}^{j}$ values. The *x*-axis and *y*-axis values vary across all plots. Green and orange ${D}^{j}$ values indicate acceptably low and material inconsistency. The threshold of 0.64 was employed

The smoking cessation network had six target comparisons (Fig. 5, Table 3 in [14]). All target comparisons were associated with statistically nonsignificant inconsistency since the 95% intervals for inconsistency included the null value. The posterior densities of the direct and indirect log ORs overlapped almost perfectly for group counselling versus self-help, yielding a low ${D}^{j}$ value at 0.03 and indicating very low average information loss and trivial inconsistency. The subsequent two target comparisons (self-help and group counselling versus no contact) exhibited a higher average information loss at 0.21 and 0.27, respectively, for having somewhat different posterior means and standard deviations; however, they covered a similar range of log ORs overall, yielding an acceptable inconsistency at the threshold of 0.64 (Fig. 5).

The direct and indirect posterior densities of individual counselling versus self-help partially overlapped since positive log ORs of the indirect estimate received the most density, whereas the direct estimate was almost centred at zero, leading to an average information loss of 0.60, very close to the threshold (Fig. 5). For the remaining two target comparisons, the direct and indirect posterior densities had poor overlap as they covered a different range of log ORs greatly (group versus individual counselling) or had substantially different probability densities for the common range (individual counselling versus no contact), resulting in substantial average information loss that greatly exceeded the threshold of 0.64, suggesting material inconsistency (Fig. 5).

The percentage contributions of ${D}_{D,I}^{j}$ and ${D}_{I,D}^{j}$ to the total information loss were less extreme than those observed in the first example (Additional file 1: Figure S2). The less the posterior densities differed in their dispersion and location, the closer to 50% were the contributions of ${D}_{D,I}^{j}$ and ${D}_{I,D}^{j}$, which is evident for the first two target comparisons. In target comparisons with acceptably low inconsistency, the ${D}_{D,I}^{j}$ and ${D}_{I,D}^{j}$ values ranged from 0.03 to 0.78 and 0.03 to 0.42, respectively, attributed to the overall sufficient overlapping of the posterior densities.

### Parkinson’s disease network (third example)

**Fig. 6:**
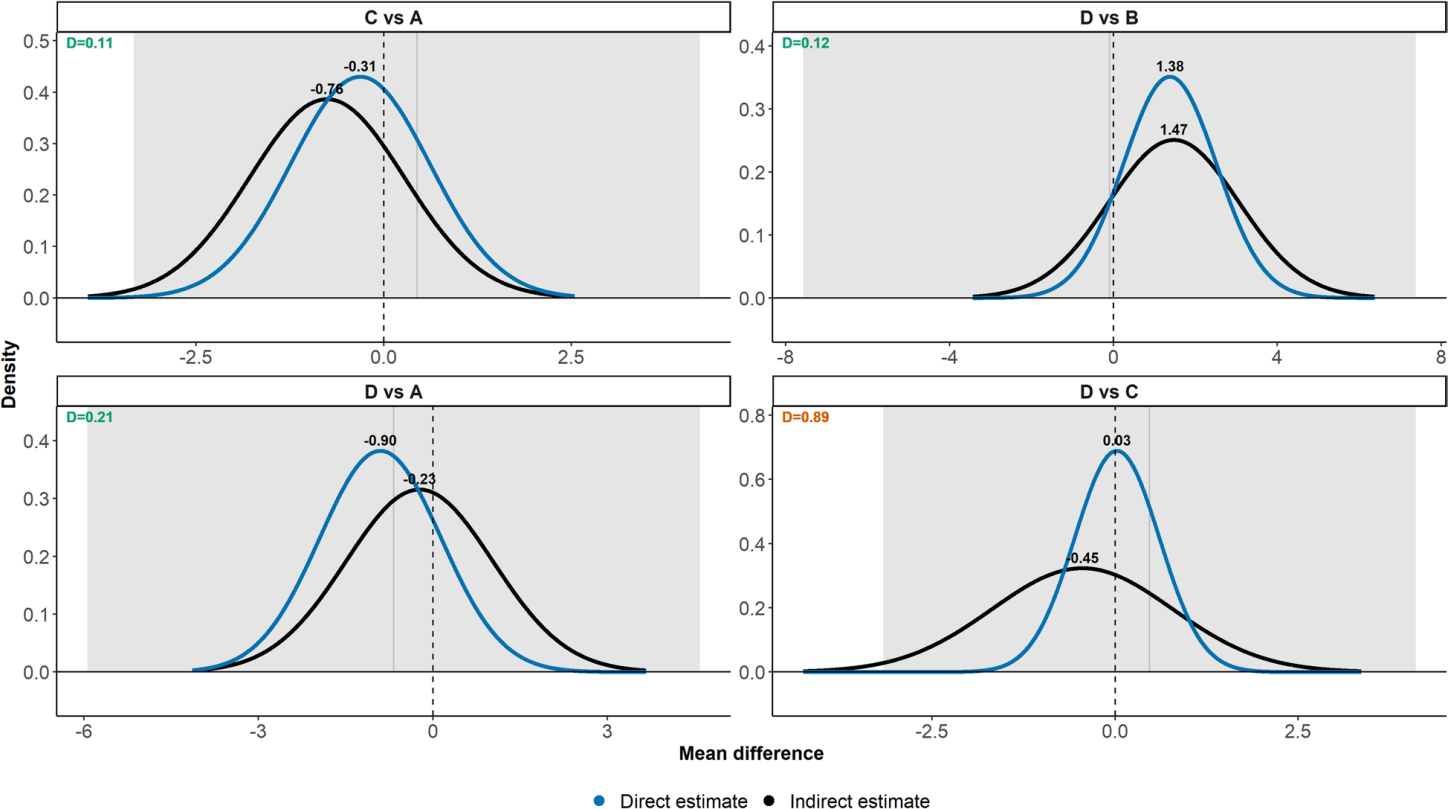
Posterior densities of the direct (blue line) and indirect (black line) MD for four target comparisons from the Parkinson’s disease network (third example). The grey area and vertical line indicate the inconsistency’s 95% credible interval and posterior mean. The average information loss (${D}^{j}$) appear at the top left of each plot. The plots have been sorted in ascending order of the ${D}^{j}$ values. The *x*-axis and *y*-axis values vary across all plots. Green and orange ${D}^{j}$ values indicate acceptably low and material inconsistency. The threshold of 0.64 was employed

Figure 6 illustrates the posterior densities of direct and indirect MDs for four target comparisons in the Parkinson’s disease network. All target comparisons were associated with statistically nonsignificant inconsistency. The target comparisons ropinirole (C) versus placebo (A) and bromocriptine (D) versus pramipexole (B) were associated with the lowest average information loss for having sufficiently overlapping probability densities, yielding ${D}^{j}$ values below the threshold of 0.64. In bromocriptine (D) versus placebo (A), the direct and indirect probability densities indicated different conclusions in the treatment preference, with the direct MD tending to favour bromocriptine over placebo; however, the posterior densities had good overlapping concerning the range of MDs, and, hence, the average information loss suggested an acceptable inconsistency at 0.21 (Fig. 6). In this target comparison, the ${D}_{D,I}^{j}$ and ${D}_{I,D}^{j}$ values were small, and their contributions to the total information loss were quite balanced (Additional file 1: Figure S3).

The posterior densities of direct and indirect MDs for bromocriptine (D) versus ropinirole (C) differed substantially in the range of MDs and their densities: the indirect MD tended to favour bromocriptine over ropinirole with enough uncertainty, but the direct MD was almost centred at zero and more precise (Fig. 6). As expected, the ${D}^{j}$ value at 0.89 suggested substantial information loss and, thus, material inconsistency. Approximating the imprecise indirect posterior density with the more precise direct posterior density led to substantial information loss (${D}_{I,D}^{j}=1.34$ versus ${D}_{D,I}^{j}=0.44$) and a 75% contribution to the total information loss for bromocriptine (D) versus ropinirole (C) (Additional file 1: Figure S3).

## Discussion

The present study proposed an intuitive framework to interpret local inconsistency based on the well-known KLD measure and a semi-objective threshold of acceptably low inconsistency. The proposed framework is straightforward to implement, as the only requisite is the results from a local inconsistency evaluation method. Quantifying the extent of distribution overlap in terms of information loss to gauge the closeness of the direct and indirect estimates is a novel contribution to the methodological framework for consistency evaluation.

In line with Dias et al. [14], in the first example, we judged the target comparison ASPAC versus Acc t-PA to have material inconsistency for having an immense ${D}^{j}$ value at 134.08, which aligned with the very low Bayesian *p*-value at 0.001 (Table 2 in [14]). In contrast to Dias et al. [14], we found many more target comparisons with material inconsistency, especially those with ${D}^{j}$ values that greatly exceeded 10. The direct and indirect distributions of these target comparisons hardly overlapped. In the second example, we found acceptably low inconsistency in four out of six target comparisons. Overall, our findings agreed with those of Dias et al. [14]; our framework revealed material inconsistency for group versus individual counselling (${D}^{j}=4.95$), which aligned with the low Bayesian *p*-value at 0.07 (Table 3 in [14]).

Lastly, our conclusions concurred with those of van Valkenhoef et al. [18] that inconsistency may not be concerning overall in the network (third example). However, we judged one comparison to be associated with *potentially* material inconsistency for having ${D}^{j}=0.89$ that slightly exceeded the threshold of 0.64. For the common range of values, the distributions differed notably in their probability densities, and we would prefer to juxtapose this target comparison with the remaining network to determine whether concerns about material inconsistency are justified.

The stochastic search inconsistency factor selection (SSIFS) is another contribution to detecting possible inconsistency in a network that deviates from the ‘standard decision-making framework’ [27]. This Bayesian approach treats the inconsistency factors as variables in a regression model and evaluates network consistency by utilising variable selection techniques [27]. Each inconsistency factor is included in the NMA model with a probability; lower inclusion probabilities of the inconsistency factors suggest a higher likelihood of network consistency [27]. SSIFS evaluates network consistency globally and locally by identifying the sources of inconsistency using posterior odds and posterior inclusion probabilities [27]. Prior knowledge regarding network consistency and practical significant differences between direct and indirect evidence can be incorporated into the inconsistency detection process, making this approach particularly attractive [27]. The authors also used the smoking cessation network to exemplify their novel approach to inconsistency, and our conclusions concurred overall.

The analysis framework of Dias et al. [14] and van Valkenhoef et al. [18] for inconsistency evaluation also differed from the ‘standard decision-making framework’. The authors employed several statistical tools and illustrations to understand to what extent consistency governed the analysed networks, offering a thorough evaluation, which served as an important reminder that inconsistency evaluation should be a multifaceted procedure, including model fit and comparison and outlier detection, apart from merely statistical testing (the current status quo) [14, 18]. Nevertheless, the authors judged the distribution overlap of the compared evidence sources based on subjective grounds, rendering our semi-objective interpretation framework a valuable aid in situations where judgements are less obvious (e.g. the target comparisons with ${D}^{j}\ge 0.64$ in Figs. 5 and 6).

The CINeMA (Confidence in Network Meta-Analysis) framework also implements a multifaceted approach to determine any concerns regarding inconsistency in a connected network, however, grounded mostly on the ‘standard decision-making framework’. Specifically, CINeMA considers the closeness of the direct and indirect estimates, their position about the clinically defined range of equivalence, and whether the *p*-value of global or local inconsistency exceeds the significance threshold to determine whether there are major, some, or no concerns with inconsistency (incoherence in the GRADE ‘language’) [28]. This framework is attractive for using clinical judgment to define the range of equivalence and being straightforward to apply. However, like Dias et al. [14], CINeMA relies on subjective judgements about the closeness of the direct and indirect estimates and their confidence intervals, which may challenge the reproducibility of the judgements. Furthermore, reliance on the *p*-value may perpetuate the misinterpretation of statistically nonsignificant inconsistency tests as evidence of consistency. Our proposed interpretation framework addresses both limitations and can be used to define thresholds that reflect the level of concern about potential inconsistency more reliably.

We proposed a semi-objective threshold based on the assumption that the variance of the normal distribution for the indirect effect is twice the variance of the (typically assumed) normal distribution for the direct effect, and the inconsistency is a function of that variance. Assuming an even larger variance proportionally would yield a much larger threshold, signalling acceptably low inconsistency more frequently. Therefore, carefully determining the threshold of acceptably low inconsistency is pivotal for the reliability of the conclusions derived from the proposed framework and ideally should incorporate clinical judgements. For instance, the analysts could replace ${\mu }_{D}-{\mu }_{I}$, ${s}_{D}$, and ${s}_{I}$ in Eqs. (1) and (2) with clinically plausible values that align with the investigated clinical field and reflect clinically unimportant inconsistency to obtain a contextualised threshold for ${D}^{j}$.

Furthermore, attentively selecting the method to evaluate inconsistency locally is also crucial to the reliability of the conclusions using our proposed framework. For instance, in networks with multi-arm studies, the loop-specific approach is unsuitable for not properly handling multi-arm studies [29]. Then, accompanying the loop-specific results with our proposed approach will more likely add ‘noise’ than value. Dias et al. [14] discussed the limitations of using the back-calculation approach in a network with multi-arm studies (the thrombolytics network), where this approach yielded quite different results from the node-splitting approach for target comparisons found in multi-arm studies. However, both approaches pointed to potential inconsistency in the network [14].

Published empirical studies have relied on the ‘standard decision-making framework’ to gauge the commonness of inconsistency [16, 30]. Given the low statistical power of the inconsistency tests, inconsistency may be more prevalent than already reported. Incorporating our interpretation approach into the analysis plan of an empirical study would more reliably capture the extent of local inconsistency as it warrants an informed decision about potential (in)consistency when statistical tests are inconclusive.

## Conclusions

The ‘standard decision-making’ approach to inconsistency assessment cannot infer consistency, as a *p*-value above the significance threshold is not evidence of consistency. Concluding treatment equivalence or consistency in a network requires a carefully designed procedure that also grants equivalence statements. The available tests for local and global inconsistency can only provide evidence of inconsistency when sufficiently powered. Consistency evaluation requires a multifaceted approach that extends beyond statistical testing. Our semi-objective interpretation framework for inconsistency is a valuable addition to the toolkit for a multifaceted inconsistency assessment as it aids in uncovering the parts of the network associated with (in)consistency when statistical tests are inconclusive by juxtaposing the information loss from the indirect and direct probability densities with a carefully selected threshold of acceptably low inconsistency.

## Supplementary Information

Additional file 1: Figure S1. Probability densities of the direct and indirect log ORs for 14 target comparisons from the thrombolytics network under the back-calculation approach. Figure S2. Bar plots with the percentage contribution of approximating direct and indirect posterior densities to their total information loss from the smoking cessation network. Figure S3. Bar plots with the percentage contribution of approximating direct and indirect posterior densities to their total information loss from the Parkinson's disease network.

## Data Availability

The data supporting the present study’s findings can be found in the cited articles. The functions related to the present study to reproduce the results are publicly available at https://github.com/LoukiaSpin/Local-inconsistency-Kullback-Leibler-divergence.git.

## Abbreviations

Acc t-PA: Accelerated alteplase
t-PA: Alteplase
ASPAC: Anistreplase
CINeMA: Confidence in Network Meta-Analysis
KLD: Kullback-Leibler divergence
MD: Mean difference
NMA: Network meta-analysis
OR: Odds ratio
PTCA: Percutaneous transluminal coronary angioplasty
r-PA: Reteplase
SK: Streptokinase
SK + t-PA: Streptokinase plus alteplase
SSIFS: Stochastic search inconsistency factor selection
TNK: Tenecteplase
UK: Urokinase
