# Supplementary material for: Local inconsistency detection using the Kullback–Leibler divergence measure
Source: Syst Rev. 2024 Oct 17;13:261. doi: 10.1186/s13643-024-02680-4 (PMC11487772; doi:10.1186/s13643-024-02680-4)

**Supporting Information for the article 'Local inconsistency detection using the Kullback-Leibler divergence measure'**

Loukia M. Spineli^1^  [Spineli.Loukia@mh-hannover.de](mailto:Spineli.Loukia@mh-hannover.de)

^1^Midwifery Research and Education Unit, Hannover Medical School, Hannover, Germany


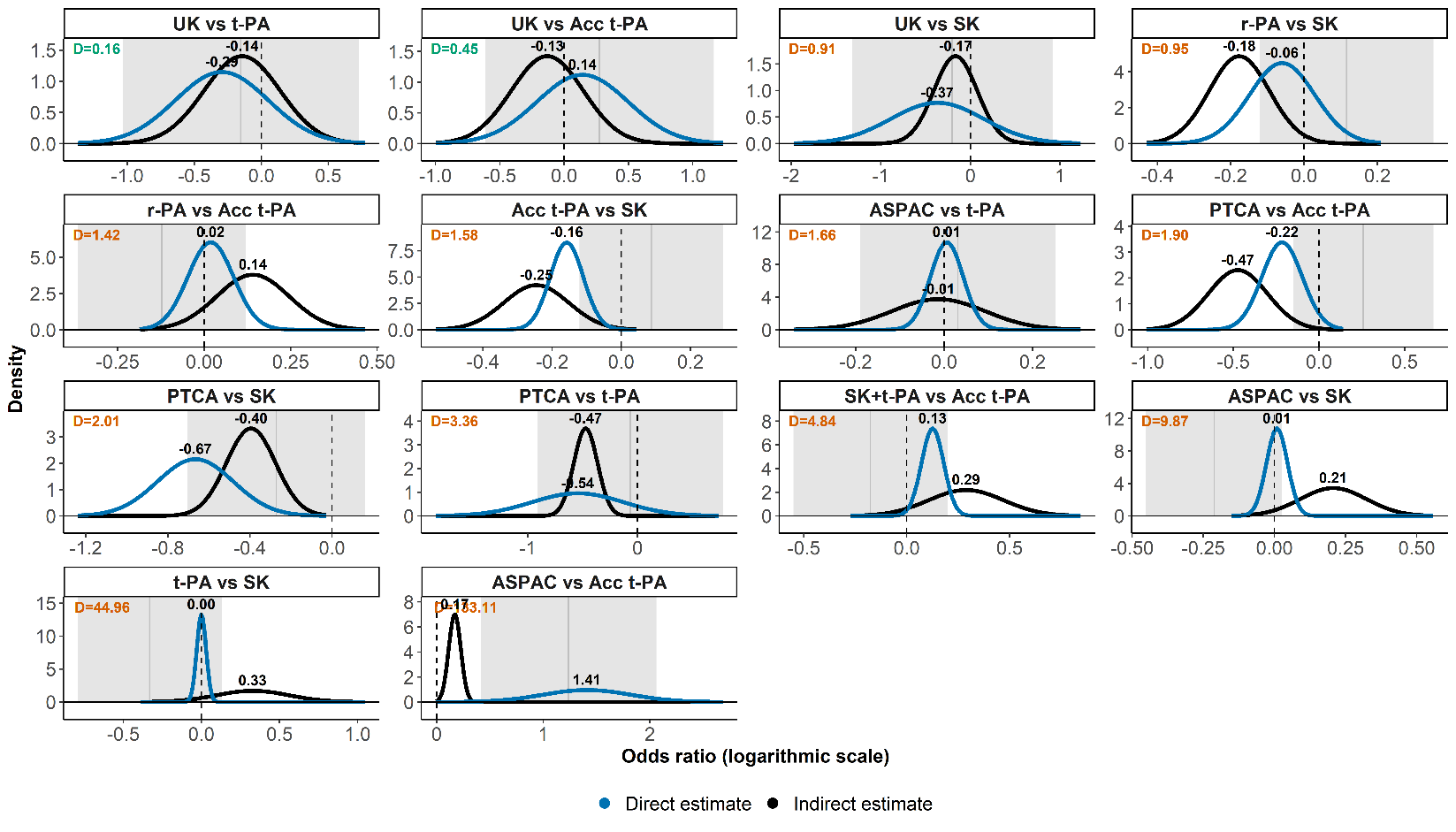


**Figure S1.** Probability densities of the direct (blue line) and indirect (black line) log ORs for 14 target comparisons from *the thrombolytics network* (first example) under the back-calculation approach. The grey areas and vertical lines indicate the inconsistencies' 95% confidence interval and mean. The average information loss ($D^{j}$) appear at the top left of each plot. The plots have been sorted in ascending order of the $D^{j}$ values. The x-axis and y-axis values vary across all plots. Green and orange $D^{j}$ values indicate acceptably low and material inconsistency. The threshold of 0.64 was employed.


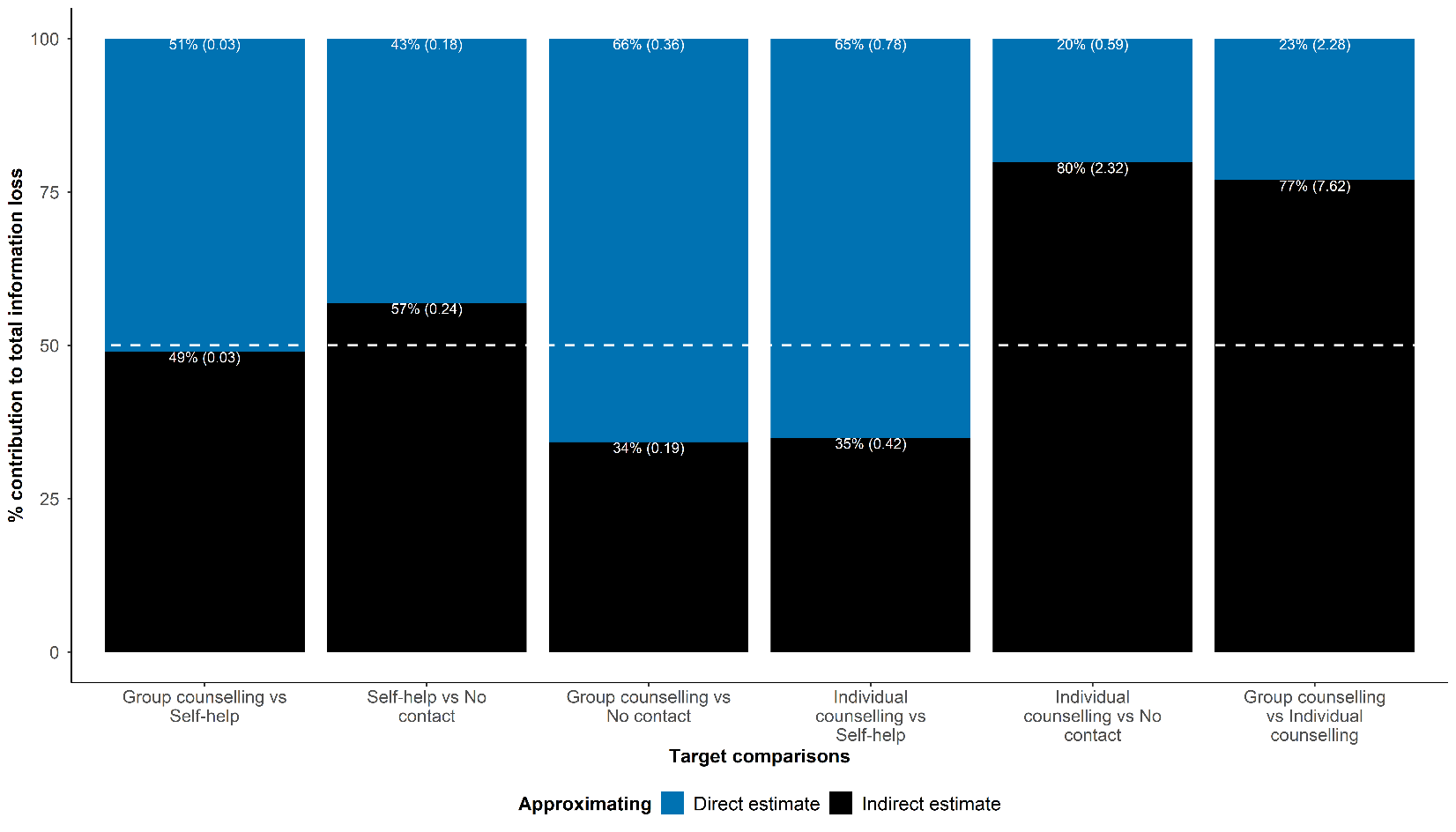
**Figure** **S2.** Bar plots with the percentage contribution of approximating direct posterior density (blue bars, $D_{D,I}^{j}$) and indirect posterior density (black bars, $D_{I,D}^{j}$) to their total information loss ($D_{D,I}^{j}+D_{I,D}^{j}$) for each target comparison (x-axis) from the *smoking cessation network* (second example). Percentage contributions appear outside the parenthesis. The plots have been sorted in ascending order of the $D^{j}$ values. The $D_{D,I}^{j}$ and $D_{I,D}^{j}$ values appear in the parentheses.

**Figure S3.** Bar plots with the percentage contribution of approximating direct posterior density (blue bars, $D_{D,I}^{j}$) and indirect posterior density (black bars, $D_{I,D}^{j}$) to their total information loss ($D_{D,I}^{j}+D_{I,D}^{j}$) for each target comparison (x-axis) from the *Parkinson's disease network* (third example). Percentage contributions appear outside the parenthesis. The plots have been sorted in ascending order of the $D^{j}$ values. The $D_{D,I}^{j}$ and $D_{I,D}^{j}$ values appear in the parentheses.
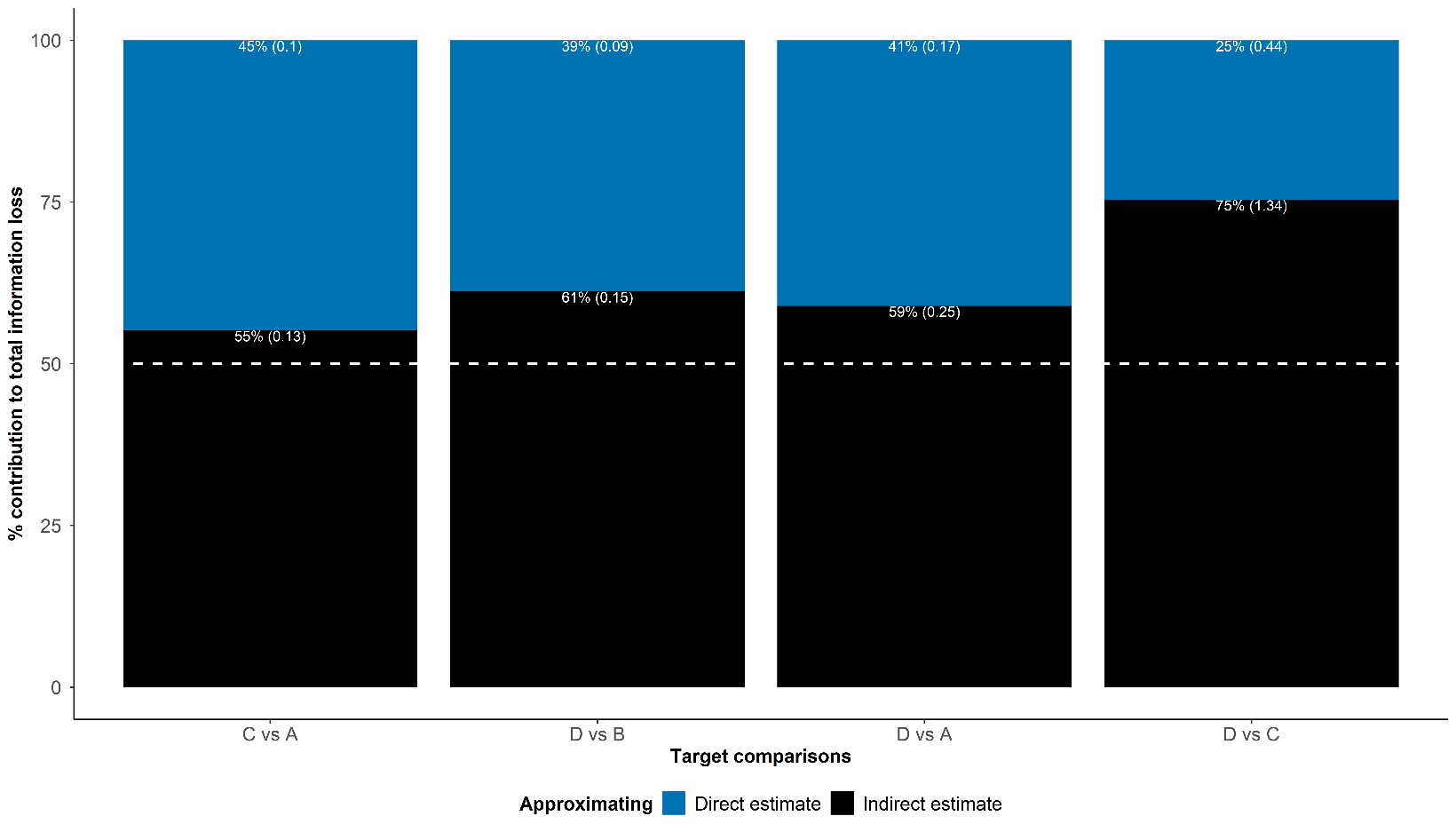

Supplement: Supplementary file 1 — Additional file 1: Figure S1. Probability densities of the direct and indirect log ORs for 14 target comparisons from the thrombolytics network under the back-calculation approach. Figure S2. Bar plots with the percentage contribution of approximating direct and indirect posterior densities to their total information loss from the smoking cessation network. Figure S3. Bar plots with the percentage contribution of approximating direct and indirect posterior densities to their total information loss from the Parkinson's disease network. [file 13643_2024_2680_MOESM1_ESM.docx]
